# Functional enhancement of neuronal cell behaviors and differentiation by elastin-mimetic recombinant protein presenting Arg-Gly-Asp peptides

**DOI:** 10.1186/1472-6750-12-61

**Published:** 2012-09-14

**Authors:** Won Bae Jeon, Bo Hyung Park, Seong Kyoon Choi, Kyeong-Min Lee, Jin-Kyu Park

**Affiliations:** 1 Laboratory of Biochemistry and Cellular Engineering, Division of NanoBio Technology, Daegu Gyeongbuk Institute of Science and Technology, Daegu 711-873, South Korea

**Keywords:** Elastin-mimetic proteins, Biomimetic matrix, Cell adhesion, Cell spreading, Cell migration, Neuronal differentiation

## Abstract

**Background:**

Integrin-mediated interaction of neuronal cells with extracellular matrix (ECM) is important for the control of cell adhesion, morphology, motility, and differentiation in both *in vitro* and *in vivo* systems. Arg-Gly-Asp (RGD) sequence is one of the most potent integrin-binding ligand found in many native ECM proteins. An elastin-mimetic recombinant protein, TGPG[VGRGD(VGVPG)_6_]_20_WPC, referred to as [RGD-V_6_]_20_, contains multiple RGD motifs to bind cell-surface integrins. This study aimed to investigate how surface-adsorbed recombinant protein can be used to modulate the behaviors and differentiation of neuronal cells *in vitro*. For this purpose, biomimetic ECM surfaces were prepared by isothermal adsorption of [RGD-V_6_]_20_ onto the tissue culture polystyrene (TCPS), and the effects of protein-coated surfaces on neuronal cell adhesion, spreading, migration, and differentiation were quantitatively measured using N2a neuroblastoma cells.

**Results:**

The [RGD-V_6_]_20_ was expressed in *E. coli* and purified by thermally-induced phase transition. N2a cell attachment to either [RGD-V_6_]_20_ or fibronectin followed hyperbolic binding kinetics saturating around 2 μM protein concentration. The apparent maximum cell binding to [RGD-V_6_]_20_ was approximately 96% of fibronectin, with half-maximal adhesion on [RGD-V_6_]_20_ and fibronectin occurring at a coating concentration of 2.4 × 10^-7^ and 1.4 × 10^-7^ M, respectively. The percentage of spreading cells was in the following order of proteins: fibronectin (84.3% ± 6.9%) > [RGD-V_6_]_20_ (42.9% ± 6.5%) > [V_7_]_20_ (15.5% ± 3.2%) > TCPS (less than 10%). The migration speed of N2a cells on [RGD-V_6_]_20_ was similar to that of cells on fibronectin. The expression of neuronal marker proteins Tuj1, MAP2, and GFAP was approximately 1.5-fold up-regulated by [RGD-V_6_]_20_ relative to TCPS. Moreover, by the presence of both [RGD-V_6_]_20_ and RA, the expression levels of NSE, TuJ1, NF68, MAP2, and GFAP were significantly elevated.

**Conclusion:**

We have shown that an elastin-mimetic protein consisting of alternating tropoelastin structural domains and cell-binding RGD motifs is able to stimulate neuronal cell behaviors and differentiation. In particular, adhesion-induced neural differentiation is highly desirable for neural development and nerve repair. In this context, our data emphasize that the combination of biomimetically engineered recombinant protein and isothermal adsorption approach allows for the facile preparation of bioactive matrix or coating for neural tissue regeneration.

## Background

Elastin-like proteins (ELPs) are recombinant biopolymers consisting of VPGXG pentapeptides (X is the guest position for any amino acid except proline) derived from the VPGVG sequence found in the natural matrix protein tropoelastin [1]. They are compatible with living cells [2] and also respond to changes in temperature, pressure, salt concentration, and pH through sol-gel phase transition [3]. Both biological and mechanical properties of ELPs can be readily tailored at the gene level to satisfy end-user applications, thus offering numerous choices for the development of cell culture matrices for tissue engineering [4]. However, ELPs containing a neutral or hydrophobic amino acid residue in the guest position X are limited in their usage for cell growth or tissue regeneration due to their low binding affinity to mammalian cells [5]. Therefore, modification of the VPGXG backbone with cell-adhesive sequences has been a major issue in developing ELP-based ECM analogues.

Neuronal cells require contact sites within their surrounding matrix, not only for initial cell attachment but also for long-term differentiation [6]. In the natural ECM environment, the cell-adhesion sequences or domains found in various types of ECM proteins provide contact sites and modulate neuronal cell behaviors through interaction with cell-surface receptors [7]. Among them, an RGD tripeptide derived from fibronectin, vitronectin, or laminin, is a potent integrin-binding ligand associated with adhesion-mediated cell migration and is involved in neurite elongation during neuronal cell differentiation [8]. An RGD ligand has been utilized to increase the cell adhesion affinity of the ELPs. For example, an APGVGV-based ELP did not attach to 3 T3-L1 fibroblasts, however, when it was fused with the RGD peptide, the resulting polypeptide was able to bind fibroblasts [9]. Similarly, the RGD-VPGIG polypeptide supports stronger spreading of HUVECs than its negative control containing a scrambled RDG sequence [10]. These RGD-functionalized ELPs have been explored in non-neural and neural cell growth, especially in a recent study on PC-12 neuronal cells that highlights the potential of RGD-incorporated ELTCPS as part of a biocompatible coating in neuronal tissue engineering [11].

An RGD-containing ELP, [RGD-V_6__20_, has previously been produced through genetic recombination of RGD ligand with VGVPG pentapeptide [12]. Preliminary biological assays showed that [RGD-V_6__20_ was not toxic with respect to the viability of fibroblasts or neuroblasts and was more effective in promoting the proliferation of neuroblasts than its counterpart that contained no RGD motifs. By using a neuroblastoma cell line, N2a, as a test model for *in vitro* assays, this study therefore aimed to further investigate the potential feasibility of this fusion protein as an ECM analogue with the ability to modulate neuronal cell behaviors and differentiation. For this purpose, biomimetic surfaces were prepared through the isothermal adsorption of [RGD-V_6__20_ on TCPS, and the effects of protein-coated surfaces on N2a cell adhesion and migration as well as on the expression of neuronal biomarkers were quantitatively measured by quantitative RT-PCR (qRT-PCR) and immunofluorescence staining.

## Results

### Purification and characterization of [RGD-V_6_]_20_

**Figure 1:**
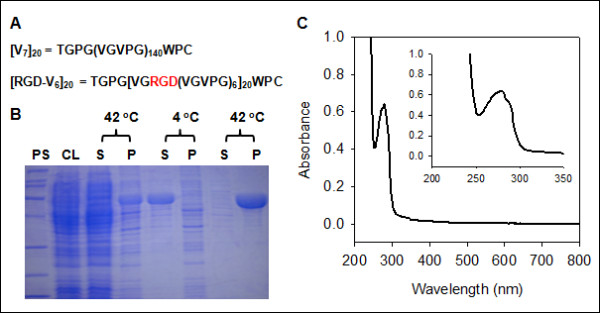
**(A) Amino acid sequences of [V**_**7**_**]**_**20**_**and [RGD-V**_**6**_**]**_**20**_**. (B) **SDS-PAGE analysis of [RGD-V_6_]_20_ purification by inverse transition cycling at 42°C and 4°C. (**C**) UV-visible spectrum of [RGD-V_6_]_20_ in PBS. PS shows MWs of protein standards from top to the bottom: 200, 116, 97, 66, 45, 31, 22 and 14 kDa. CL, S and P represent cell lysate, supernatant and pellet, respectively.

The primary structures of [V_7_]_20_ and are shown in Figure 1A. In [RGD-V_6_]_20_, 20 RGD motifs were evenly distributed throughout the whole molecular structure. Typically, from a 40 l fermentation batch of *E. coli*, approximately 600 g of wet cell cake was obtained. After 3 rounds of inverse transition cycling, 1,036 and 1,080 mg of [V_7_]_20_ and [RGD-V_6_]_20_, respectively, were recovered from a 40 liter batch with a purity greater than 95%, as judged by densitometry on an SDS-PAGE gel (Figure 1B). When a UV-visible spectrum of [RGD-V_6_]_20_ in phosphate-buffered saline (PBS) was scanned from 800 to 200 nm (Figure 1C), its maximum absorbance occurred at 280 nm, and 2 shoulders were detected at 273 (*ε* = 5,505 M^-1^ cm^-1^) and 288 nm (*ε* = 4,666 M^-1^ cm^-1^). No absorbance was detected in the visible region.

### Measurement of cell adhesion affinity

To investigate the effect of RGD modification on cell adhesion, a series of comparative assays was performed on the surfaces of TCPS, [V_7_]_20_, [RGD-V_6_]_20_, and fibronectin. As an initial test, protein-coated surfaces were prepared by isothermal adsorption of 5 μM protein solutions at 4°C.

**Figure 2:**
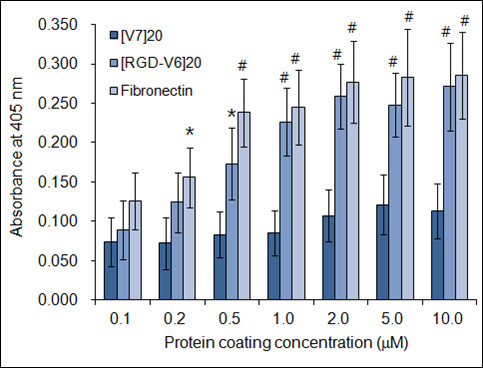
**Dependence of N2a cell adhesion on protein coating concentration. **Protein-coated surfaces were prepared using 0.1, 0.2, 0.5, 1, 2, 5 or 10 μM of [V_7_]_20_, [RGD-V_6_]_20_ and fibronectin. Protein concentration was prepared based on the calculated MWs of 58.2, 59.6 and 225 kDa for [V_7_]_20_, [RGD-V_6_]_20_ and fibronectin. A_405_ values of samples were subtracted by 0.035 ± 0.004 of background control and used to generate cell adhesion graphs. * and # correspond to P < 0.05 and P < 0.01 from [V_7_]_20_, respectively.

The relative percentages of cell attachment on TCPS, [V_7_]_20_, and [RGD-V_6_]_20_ were 12% ± 2.8%, 43% ± 7.4% and 88% ± 9.5% of fibronectin, respectively, suggesting efficient cell adhesion to the [RGD-V_6_]_20_ coated surface. To determine the cell-binding constants, the plots of cell adhesion versus protein-coating concentration were calculated and are displayed in Figure 2. As the coating concentration increased from 0.1 to 10 μM, no noticeable cell-binding pattern was observed on the plot for [V_7_]_20_; the data did not fit to either a linear first order (r^2^ = 0.582) or hyperbolic (r^2^ = 0.816) regression. In contrast, N2a adhesion to either [RGD-V_6_]_20_ (r^2^ = 0.979) or fibronectin (r^2^ = 0.980) resulted in a hyperbolic graph with a saturation around 2 μM protein concentration. The apparent maximum cell binding to [RGD-V_6_]_20_ was approximately 96% of fibronectin, with half-maximal adhesion on [RGD-V_6_]_20_ and fibronectin occurring at a coating concentration of 2.4 × 10^-7^ and 1.4 × 10^-7^ M, respectively.

The saturated adsorption density of the RGD-VPGIG polypeptide (31 kDa) and fibronectin (225 kDa) were adopted from the published literature as being 30 ng/mm^2^ (1 pmol/mm^2^) [11] and 8 ng/mm^2^ (36 fmol/mm^2^, as monomeric form) [13], respectively. Based on the report that ELPs with different amino acid content showed a similar levels of adsorption [11], we numerically estimated the saturated surface density of [RGD-V_6__20_ as 500 fmol/mm^2^, which in turn led us to interpret that both the *B*_max_ and *K*_d_ values of [RGD-V_6__20_ for N2a cell binding would be 14-fold lower than those respective values for fibronectin.

### Stimulation of cell spreading

**Figure 3:**
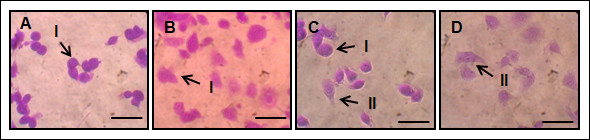
**Cell spreading morphology dependence of N2a cells on TCPS (A), [V**_**7**_**]**_**20**_**(B), [RGD-V**_**6**_**]**_**20**_**(C) and fibronectin (D). **Two cell shape classes were determined from phase-contrast microscopic images: round (I) and extended (II) shapes. Protein coating concentration was 20 μg/ml. Scale bars represent 10 μm.

The effect of protein-coated surfaces on cell spreading morphology was inspected by phase contrast microscopy. Morphological characteristics of N2a cells were divided into two shapes: round shape and extended shape. Round cells were dominant on both TCPS and (Figure 3A and B), whereas almost equal populations of two classes of cell shapes were observed on the microscopic images of the cells adhered on [RGD-V_6_]_20_-adsorbed substrates (Figure 3C). For fibronectin-coated substrate, extended cells were dominant and relatively small population of less spread cells was observed (Figure 3D). Consequently, the percentage of cell spreading increased in the following order of proteins: TCPS (less than 10%) < [V_7_]_20_ (15.5% ± 3.2% < [RGD-V_6_]_20_ (42.9% ± 6.5%) < fibronectin (84.3% ± 6.9%).

### Acceleration of cell migration speed

**Figure 4:**
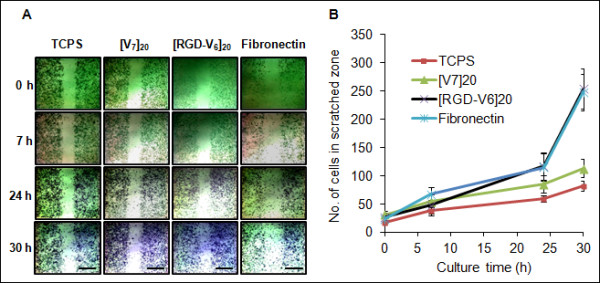
**Typical images of scratch migration assay (A) and N2a population motility (B) on TCPS, [V**_**7**_**]**_**20**_**, [RGD-V**_**6**_**]**_**20**_**and fibronectin. **Protein matrices were prepared at 20 μg/ml concentration. The color in Figure B is arbitrarily chosen and does not depict the actual color.

An N2a migration assay was performed on TCPS- and protein-coated surfaces prepared at 20 μg/ml coating concentration. The typical images of cell migration across the scratched zone are shown in Figure 4A, and the number of cells that migrated during the 30 h culture period has been compared in Figure 4B. When cells were grown on TCPS and [V_6_]_20_, the number of cells in the scratched area steadily increased over the 30-h period, giving CMI values of (2.1 ± 0.4)/h and (2.7 ± 0.7)/h, respectively. When cell motility on [RGD-V_6_]_20_ and fibronectin was measured after 24 h of culture, the N2a cells migrated with the same speed of (3.8 ± 0.6)/h, which was 1.8-fold faster than the mobility of the cells on TCPS. However, when the number of migrated cells were counted at 30 h of culture, the CMI was significantly (P < 0.01) enhanced to (7.5 ± 0.7)/h. Interestingly, during the 24–30 h culture period, the CMI values measured for [RGD-V_6_]_20_ and fibronectin were (22.8 ± 2.7)/h and (22.2 ± 1.8)/h, respectively, which were approximately 8-fold faster than the migration speed of the cells on the [V_7_]_20_-coated surface during the same period.

### Up-regulation of neuronal biomarker expression

**Figure 5:**
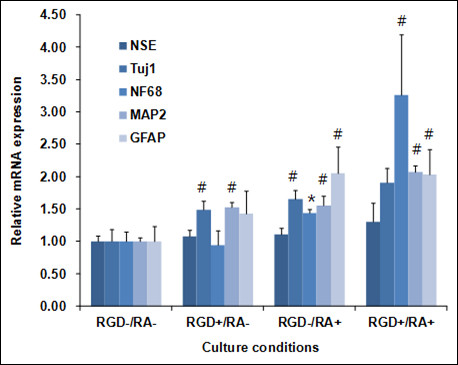
**Comparison of the mRNA levels of neuronal biomarkers expressed in N2a cells cultured on TCPS and [RGD-V**_**6**_**]**_**20**_**in the presence or absence of RA. **GAPDH mRNA was used as an internal standard. All the data are expressed as fold increase relative to the value obtained with TCPS without RA. * and # correspond to P < 0.05 and P < 0.01 compared to RGD-/RA- data, respectively.

We evaluated whether [RGD-V_6__20_ could affect N2a differentiation by measuring the expression levels of several neuronal proteins such as NSE and Tuj1, NF68, MAP2, and GFAP, which have been widely used as biomarkers for neuronal cells, axonal development, dendritic cells, and astrocytes, respectively [14]. Figure 5 compares the mRNA levels of these markers in N2a cells grown on TCPS or [RGD-V_6__20_ in the presence and absence of retinoic acid (RA). The expression of TuJ1 (1.5-fold), MAP2 (1.5-fold), and GFAP (1.4-fold) was enhanced significantly (P < 0.05) in the cells cultured on [RGD-V_6__20_, whereas the expression of NSE and NF on [RGD-V_6__20_ did not differ from that of the cells grown on TCPS. The mRNA levels of marker proteins were further elevated following RA treatment, the highest expression being observed in the cells cultured on [RGD-V_6__20_ treated with RA.

### Promotion of neuronal differentiation along with neurite outgrowth

**Figure 6:**
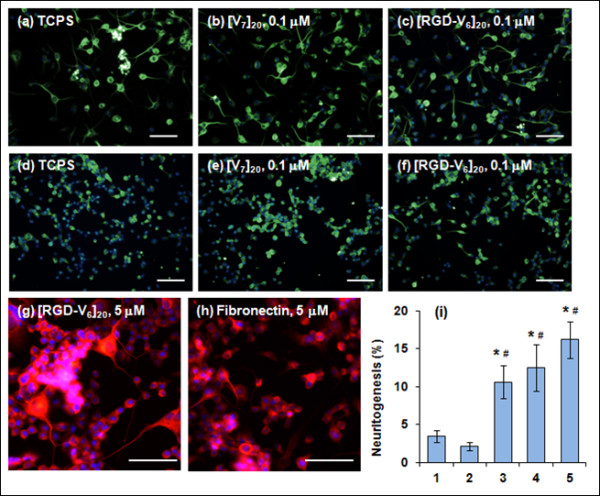
**Immunofluorescence analyses of the effect of [V**_**7**_**]**_**20**_**-, [RGD-V**_**6**_**]**_**20**_**-, and fibronectin-coated surfaces on the N2a differentiation into TuJ1-positive neurons.** N2a cells were cultured in the presence of 20 μM RA treatment (**a**-**c**) or absence of RA (**d**-**h**). Protein coating concentration was 0.1 μM (**b**, **c,e** and **f**) or 5 μM (**g** and **h**). Green and red colors originate from the Alexa Fluor 488-labeled and Alexa Fluor 568-conjugated anti-TuJ1 antibody, respectively. Blue color corresponds to DAPI. Scale bar is 50 μm. Graph (**i**) compares the percentages of neuritogenesis on TCPS (1), 0.1 μM [V_7_]_20_ (2), 0.1 μM [RGD-V_6_]_20_ (3), 5 μM [RGD-V_6_]_20_ (4) and 5 μM fibronectin (5). * and # represent P < 0.05 and P < 0.01 from TCPS and [V_7_]_20_, respectively.

Immunofluorescence staining of Tuj1 expression was performed to further validate the stimulatory effect of [RGD-V_6_]_20_ on neuronal differentiation. Figure 6 shows the immunofluorescence images of N2a cells on TCPS, [V_7_]_2_, [RGD-V_6_]_20_, and fibronectin after 5 days of culture. Initially, culture surfaces were prepared using 0.1 μM of protein solutions. In response to 20 μM RA treatment, most N2a cells extended neurites on the TCPS, [V_7_]_20_, and [RGD-V_6_]_20_ surfaces (Figure 6a, b, c). In the absence of RA, less than 4% of cells showed neurite formation on TCPS and [V_7_]_20_ (Figure 6c, d), whereas on [RGD-V_6_]_20_, 10.6% ± 2.2% (P < 0.05) of cells exhibited tadpole-like morphological features with sprouting neurites (Figure 6f). We subsequently analyzed the neuritogenesis at 5 μM coating concentration, and the percentage of neuritogenesis was found to be 12.5% ± 3.1%, which was significantly different from the values obtained for TCPS and [V_7_]_20_ (P < 0.05) but was not statistically significant (P > 0.05) from the value obtained at the 0.1 μM [RGD-V_6_]_20_ coating concentration. Interestingly, N2a cells bearing 2–4 nuclei within a single cytosol body with multiple neurites were seen (Figure 6g). In parallel experiments, the effect of fibronectin on N2a differentiation was analyzed at the 5 μM coating concentration, and 15.2% ± 2.4% of neurite formation was noted (Figure 6h). Figure 6i compares the neuritogenesis of N2a cells on TCPS and protein-coated surfaces and clearly shows that [RGD-V_6_]_20_ promotes neuronal differentiation.

## Discussion

An ELP-based recombinant protein has been produced to develop a bioactive matrix that can stimulate the neuronal cell behaviors and differentiation. To evaluate biological effects of RGD-modified ELP on neural cell behaviors and particularly on neural differentiation *in vitro*, bioactive substrates were prepared by physically adsorption of the RGD-modified recombinant ELPs onto the conventional TCPS. Isothermal adsorption process has been known as a robust method to generate biomimetic surfaces suitable for displaying and investigating the effects of ECM functional domains on neural differentiation [11].

Cell adhesion affinity of [RGD-V_6__20_ was approximately 2-fold higher than that of [V_7__20_. The mode of N2a binding to [RGD-V_6__20_ followed hyperbolic saturation and was similar to the adhesion of the cells on fibronectin-coated surface, where cell attachment reached a plateau at the high surface density of the RGD peptide. Both the enhanced attachment of the cells onto [RGD-V_6__20_ in comparison with that on [V_7__20_ and saturable binding mode are indicatives of RGD-mediated cell binding through cell-surface receptors. Based on the numerically estimated saturation values, the difference in maximum cell adhesion capacity between [RGD-V_6__20_ and fibronectin would fall within 2 orders of magnitude. N2a cells were found to have a higher affinity for [V_7__20_ than for TCPS control surfaces. This is similar to the enhanced interaction observed between fibroblasts and X20-poly(GVGVP), but only if FBS was added to the assay media [5]. However, throughout this study, [V_7__20_ was inefficient in stimulating N2a cell migration and differentiation, indicating that the interaction of N2a cells with [V_7__20_ is physiologically irrelevant. Therefore, attachment of N2a cells to the [V_7__20_-coated surfaces would possibly be mediated by cell-adhesion molecules originated from the FBS, as shown by the fibroblast adhesion to the poor-cell-binding X20-poly(GVGVP) [5].

The effect of [RGD-V_6__20_ coatings in N2a cells spreading experiments showed a similar cellular morphology to that seen on fibronectin-coated surfaces, which seems to correlate with enhanced cell spreading. The result presented in this study is in good agreement with previous report whereby fibroblasts formed spreading structures such as lamellipodia and filopodia at the edges of the cells grown on the [RGD-V_6__20_ matrix [12].

On the cell-adhesive surfaces, cell locomotion is governed by the interactions between the integrin receptors and their ligands displayed on the cell surfaces [15]. In comparison with the motility on [V_7__20_, which lacks integrin-adhesion ligands, N2a cells migrated much faster on [RGD-V_6__20_, and therefore, this was attributed to the RGD-mediated cell adhesion. Until 24 h of culture, the number of cells that migrated across the scratched edges was not statistically different among all 4 substrates. However, during the 24–30 h culture period, a significant increase in the population of migrating cells was measured on both [RGD-V_6__20_ and fibronectin but not on TCPS and [V_7__20_. The result suggests a 2-phase migration mode, that is, initially slow movement followed by a fast migration stage, and also implies that [RGD-V_6__20_ activate signaling events within the cells to express a motile phenotype during the slow migration period.

The incorporation of RGD motifs into the relatively inert elastic VGVPG backbone has yielded an ECM analogue with fibronectin-like function in directing neural differentiation. For instance, immunofluorescence analysis of Tuj1 expression showed that [RGD-V_6__20_ promote neuronal differentiation along with neurite extension even without RA treatment. Unlike [RGD-V_6__20_, the [V_7__20_ was not able to promote neurite elongation, and the lack of neurite outgrowth on the matrix is possibly due to the absence of the RGD motifs that are necessary for proper neurite elongation [16]. Thus, enhanced neurite elongation from N2a cells reflects the activation of signal transduction at the N2a cell-[RGD-V_6__20_ interfaces. The RGD motif of fibronectin binds αvβ3and α5β1 integrins to promote neuritogenesis by PC12 cells [16,17]. Whereas, interaction of integrin α8β1 with RGD sequence in cell adhesion molecule stimulates neurite outgrowth from dorsal root ganglion [18]. In PC12 cells, RGD-mediated adhesion triggers many signaling events including focal adhesion kinase phosphorylation and cytoskeletal reorganization [17,18]. Whether these signaling cascades occur in response to [RGD-V_6__20_ remains to be studied.

The spontaneous increase in Tuj1 expression under non-differentiating conditions was in contrast to the effect of the poly(RGD-VPGIG) peptide, which supports neurite extension from PC-12 cells when nerve growth factor is present in the growth media [11]. However, up-regulation of the neuronal markers TuJ1, MAP2, and GFAP in the cells grown on [RGD-V_6__20_ confirmed that [RGD-V_6__20_ is effective in stimulating neuronal cell differentiation. Moreover, the expression levels of NSE, TuJ1, NF68, MAP2, and GFAP were significantly elevated in the presence of both [RGD-V_6__20_ and RA. This additive effect indicates that [RGD-V_6__20_ enhances the differentiation response of neuronal cells to RA. This is important for eventual *in vivo* application of matrix protein in combination with differentiation-stimulating agents.

## Conclusions

Our results prove the feasibility of employing a genetically engineered biomimetic matrix protein for functional activation of neuronal cell behaviors. Adhesion affinity, spreading morphology, and migration speed of N2a cells on the [RGD-V_6_]_20_ protein were similar to those seen on fibronectin. Moreover, neuritogenesis and up-regulation of neuronal mark proteins have been achieved by culturing N2a cells on [RGD-V_6_]_20_-coated surfaces. Adhesion-mediated neural differentiation is highly desirable property in neural development and nerve repair. Therefore, this ELP-based ECM analogue can be used as a bioactive matrix for neural tissue engineering.

## Methods

### Expression, purification and characterization of recombinant ELP

ELPs were expressed from pET-25b(+)-1 containing the [V_7__20_ or [RGD-V_6__20_ gene in 40 l culture of *E. coli* BLR(DE3) (Novagen). Protein expression was induced at an OD_600_ of about 0.6 with 1 mM *β*-isopropyl thiogalactoside (Sigma) and allowed to express for 4 h post induction. The ELPs were by phase transition cycling [19]. The purified ELPs were dissolved in phosphate-buffered saline (PBS, pH 7.2), and their purity was analyzed by visualization on a 12% sodium dodecyl sulfate-polyacrylamide gel electrophoresis (SDS-PAGE) gel using Coomassie Blue staining. SDS-PAGE gel images were captured on an IQuant Capture 350 system and analyzed by ImageQuant TL 9 (GE Health Care). The protein concentration was determined by UV spectrophotometry using the molar extinction coefficient 5,690 M^-1^ cm^-1^ of a single Trp residue at 280 nm. The molecular weights (MWs) of [V_7__20_ or [RGD-V_6__20_ were computed by the Compute pI/MW software available from the ExPASy Proteomics Server and were 59,545 and 58,044 Da, respectively. Throughout this study, all experiments including biological assays were carried using the non-reduced, as-purified form of ELPs [12].

### Cell culture and maintenance

The N2a cell line (ATCC CCL-131) was used as a test model for *in vitro* neuronal cell culture. N2a cells were maintained as a monolayer in EMEM medium (Gibco) supplemented with 10% (w/v) FBS, 2 mM glutamine, 1 mM sodium pyruvate, 1.5 g/l sodium bicarbonate, 100 unit/ml penicillin, and 100 μg/ml streptomycin at 37°C in a humidified atmosphere of 95% air and 5% CO_2_. For biological assays, N2a cells at 60–80% confluence were plated out in specified culture plates at a density of 10^4^–10^6^ cells per well.

### Cell adhesion assay

Cell adhesion was measured by a hexosaminidase activity assay as described previously [20]. Wells in a 96-well polystyrene plate (SPL Life Science) were treated with 100 μL of [V_7__20_, [RGD-V_6__20_, and fibronectin (R&D Systems) solutions (at concentrations of 0.1, 0.2, 0.5, 1, 2, 5, or 10 μM) at 4°C. After overnight protein adsorption, the wells were rinsed 3 times with 100 μl PBS (pH 7.2, Gibco) and blocked with 100 μl 0.5% heat-inactivated (60°C for 1 h) BSA for 1 h at 37°C. The cells were treated with trypsin and suspended in the culture media at a density of 3 × 10^5^ cells/ml; 100 μL of the cell suspension was then added to each well and incubated with 100 μl EMEM containing 2% FBS for 30 min. After incubation for 1 h at 37°C, the medium was removed, and the wells were rinsed twice with PBS to remove unattached cells. After rinsing, attached cells were incubated with 50 μl citrate buffer (50 mM, pH 5.0) containing 3.75 mM p-nitrophenyl-N-acetyl-*β*-D-glucosaminide (Sigma) and 0.25% Triton X-100 for 30 at 37°C. The reaction was stopped by adding 50 μl glycine buffer (50 mM, pH 10.4) containing 5 mM EDTA, and the absorbance was measured at 405 nm in a Titertek microplate reader. The adsorption data were fit to a Langmuir model for single species adsorption to a single site on the substratum, A = A_max_C/(*K*_d_ + C), where A is the absorbance at 405 nm, C is the coating concentration, A_max_ is the maximum absorbance, and *K*_d_ is the coating concentration required to achieve half-maximal absorbance [13].

### Cell spreading analysis

A 96-well plate was coated with 100 μl of the protein solution (20 μg/ml) at 4°C overnight and blocked with 100 μl of heat-inactivated 0.5% BSA for 1 h at 37°C. N2a cells were seeded (2 × 10^4^ cells/well) and incubated in 100 μl EMEM containing 10% FBS at 37°C in a 5% CO_2_ incubator for 24 h. After removing the supernatant, the wells were washed 3 times with 200 μl PBS, and the adherent cells were fixed and stained in 1 step with 500 μl of 0.03% crystal violet (w/v) in 20% methanol for 10 min. Using a Nikon Ts-100 light microscope, phase contrast images (at least 20 cells in an image) were taken from 3 different cell locations for each experimental sample. The number of spreading cell population was determined by manual counting of the cells showing extended cell body, i.e., ratio of long to short diameter is greater than 2. And cells that exhibited lamellipodia were also scored as spread cells. Percentage of spreading cells was defined as (spreading cells/total number of adherent) X 100.

### Cell population migration assay

A scratch assay was used to measure cell population motility [21]. The wells in a 6-well plate were treated with 2 ml of the protein solutions (20 μg/ml) at 4°C overnight and were blocked with 0.5% heat-inactivated BSA at 37°C for 1 h. N2a cells (5 × 10^5^ cells/well) were seeded and incubated with 1.2 ml EMEM supplemented with 10% FBS for 24 h at 37°C. After removing the FBS media and washing the adherent cells with 2.0 ml PBS, a cell-free gap was created by a scratch to the confluent cell layer by using a sterile Gilson 1 ml pipette tip. Three non-overlapping regions of the scratched area were taken at 0 h by using a Nikon EcliTCPSe TE 300 microscope. The cells were incubated at 37°C in EMEM containing 0.5% FBS with mitomycin C (10 μg/ml, Sigma) to block proliferation. Phase contrast microscopic images of each scratched zone were taken at a designated time during the 30 h culture period. Cell migration index (CMI) was defined as [(N_t2_ - N_0_) - (N_t1_ - N_0_)]/(t_2_ - t_1_), where N_t2_ and N_t1_ are the number of cells detected in the scratched zone at 2 different culture times, and N_0_ is the number of cells at 0 h. The number of cells in the scratched area was determined by manual counting of the spots in the microscopic images.

### Quantitative RT-PCR analysis of neuronal biomarker expression

A 12-well plate was coated with 500 μl [RGD-V_6_]_20_ (10 μM or 600 μg/ml) at 4°C overnight. After removing the protein solution, wells were washed twice with PBS. N2a cells were seeded (5 × 10^5^ cells/well) and incubated at 37°C in a 5% CO_2_ incubator for 48 h in 3 ml EMEM containing 10% FBS. Total RNA was isolated from the conditioned N2a cells using Trizol Reagent (Invitrogen). cDNA was synthesized from 4 μg of total RNA by using a High-Capacity cDNA reverse transcription kit according to the manufacturer’s instructions. qRT-PCR was performed using a SYBR Green PCR master mix kit (Applied Biosystems) in an ABI 7500 Real Time PCR System. The cycling conditions were as follows: 50°C for 2 min; 95°C for 10 min; 40 cycles of 95°C for 15 s and 1 min at 60°C. The primer sets were designed using the Primer Express 3.0 software based on the sequences from GenBank, and are shown in Table 1. The housekeeping gene GAPDH was used as an internal standard. Reaction specificity was confirmed by melting curve analysis.

**Table 1:** Primer sequences used for qRT-PCR analysis of neuronal maker proteins

**Neuronal markers**	**Forward (5**^**′**^** → 3**^**′**^**)**	**Reverse (5**^**′**^** → 3**^**′**^**)**	**GenBank accession no.**
NSE	AACGCGGGAACATCTCATTC	CGAGGTGTTCTGGGTGACTTG	BC_031739
Tuj1	ACCCCGTGGGCTCAAAAT	CCGGAACATGGCTGTGAACT	NM_023279
NF68	GGTAGCCGCCATCAGCAA	CACGCGCTCGATGAAGCT	BC_029203
MAP2	CCTGGTGCCCAGTGAGAAGA	GTCCGGCAGTGGTTGGTTAA	BC_051410
GFAP	GCTGGAGGGCGAAGAAAAC	GCCTTCTGACACGGATTTGG	BC_139358
GAPDH	AGGTTGTCTCCTGCGACTTCA	CAGGAAATGAGCTTGACAAAGTTG	NM_008084.2

### Immunofluorescence analysis of TuJ1 expression and neurite extension

Lab-Tek chamber slides were treated with 100 μl [V_7_]_20_ and [RGD-V_6_]_20_ (0.1 or 5 μM) and 5 μM fibronectin solutions at 4°C overnight. The culture plate was rinsed 3 times with 200 μl PBS twice and was blocked with 100 μl of 0.5% heat-inactivated BSA for 1 h at 37°C. N2a cells were plated at a density of 1 × 10^4^ cells per well in EMEM containing 10% FBS and was then cultured for 5 days. The cells were fixed with 4% (v/v) paraformaldehyde for 15 min at room temperature, washed 3 times in PBS buffer, permeabilized in 0.3% Triton X-100 (v/v) for 10 min, and then washed with PBS buffer. Non-specific binding was blocked with 5% normal goat serum for 30 min. The cells were then incubated with anti-tubulin beta III isoform at a 1:100 (v/v) dilution for 2 h at room temperature. After 3 washes in PBS, cells were incubated in Alexa Fluor 488- or 568-conjugated anti-mouse IgG secondary antibody (Invitrogen) for 2 h at room temperature in the dark. Nuclear sections were counterstained with DAPI (Sigma). The preparations were then mounted in Prolong Gold antifade reagent (Invitrogen). Using a Leica DMI 3000 fluorescence microscope, fluorescence images (at least 100 cells in an image) were taken from 3 different cell locations for each experimental sample. The percentage of neurite formation was determined by manual counting of the cells bearing neurites longer than the cell’s diameter.

### Statistical data analysis

Data are expressed as mean ± S.D obtained from at least three independent experiments. The statistical significance of the differences between groups was determined by one-way analysis of variance (ANOVA) and the Tukey post-hoc test. All statistical analyses were performed using GraphPad InStat (Ver. 3.05). Statistical significance was set at P < 0.05 or P < 0.01.

## Abbreviations

CMI: Cell Migration Index; NSE: Neuron Specific Enolase; TuJ1: Neuronal class III β-tubulin; NF68: Neurofilaments 68; MAP2: Microtubule-Associated Protein 2; GFAP: Glial Fibrillary Acidic Protein; GAPDH: Glyceraldehyde-3-Phosphate Dehydrogenase; DAPI: 4′,6-Diamidino-2-Phenylindole.

## Competing interests

The authors declare that they have no competing interests.

## Authors' contributions

WBJ constructed and characterized recombinant proteins, designed experiments, and wrote the paper. BHP performed the biological assays and immunofluorescence analysis. SKC, KML, and JKP carried out the qRT-PCR measurement of neuronal biomarkers. All authors reviewed, revised and approved the final manuscript.
